# Lack of effect of a parent‐delivered early language intervention: Evidence from a randomised controlled trial completed during COVID‐19

**DOI:** 10.1002/jcv2.12279

**Published:** 2024-09-14

**Authors:** Kelly Burgoyne, Stephanie Hargreaves, Nasima Akhter, Helen Cramman, Paivi Eerola, Jochen Einbeck, Vic Menzies

**Affiliations:** ^1^ Manchester Institute of Education University of Manchester Manchester UK; ^2^ Department of Anthropology Durham University Durham UK; ^3^ School of Life and Health Sciences Teesside University Middlesbrough UK; ^4^ School of Education Durham University Durham UK; ^5^ Department of Mathematical Sciences Durham University Durham UK

**Keywords:** early intervention, education, home learning environment, language, parents, RCT design, school readiness

## Abstract

**Background:**

Parents play a key role in their child's early development but evidence that parental engagement strategies are effective is unclear. The current study evaluated a parent‐delivered early language teaching programme that aimed to support children's early language and literacy skills.

**Methods:**

A multisite, pupil‐level randomised controlled trial was conducted with 450 3–4‐year‐old children and their families, recruited from 47 nurseries across Greater Manchester and Lancashire (UK). Families were randomly allocated to either the programme group (*N* = 225) who delivered an early language teaching programme for 20‐min a day, 5 days a week, for 30‐weeks or to a control group (*N* = 225) who received a box of children's books at the end of nursery. A language latent variable formed the primary outcome, which was used to assess whether the programme improved children's language and literacy skills.

**Results:**

COVID‐19 disrupted the trial, including delivery of the intervention and post‐test data collection. Data from assessments completed 10‐months after intervention showed no evidence that the children receiving language intervention had greater language skills than the control group. Similarly, no group differences were found on measures of the Home Learning Environment or school readiness.

**Conclusions:**

Whilst disruptions caused by COVID‐19 are likely to have impacted on the findings, this study nonetheless adds to the literature which suggests that parent‐delivered interventions alone may not necessarily lead to changes in home learning or to gains in children's language skills.

**Key points**

- Whilst there is strong evidence for the effectiveness of school‐based early language intervention, existing evidence for the impact of parent‐delivered intervention on child outcomes is currently limited.
- Data from a large scale RCT showed no evidence that a parent‐delivered oral language teaching programme led to gains in child language, school readiness or the Home Learning Environment.
- The findings fail to replicate previous evidence that parent‐delivered teaching is causally related to child outcomes and suggest context is critical to understanding the success of such programmes.
- Further studies should seek to identify the contextual factors which influence effectiveness of parent‐delivered interventions.

## INTRODUCTION

Early language skills play a central role in the development of a broad range of cognitive and social‐emotional abilities. Language skills provide a critical foundation for formal education (Roulstone et al., 2011) including the development of literacy (e.g., Muter et al., 2004) and numeracy (e.g., Purpura et al., 2017). Language skills also underpin social‐emotional development and behaviour (Clegg et al., 2015; Goh et al., 2021; Morgan et al., 2015). Poor language and communication skills can therefore have considerable negative consequences for educational achievement and employability, as well as mental health and social outcomes across the lifecourse (Beard, 2017; Gross, 2018). Further, language difficulties are relatively common, affecting 7.6% of children (Norbury et al., 2016), and disproportionately affect children who are socially disadvantaged (Law et al., 2017). Given the high prevalence of language difficulties and their potential negative consequences, there is a clear need for strategies to promote early language learning.

Children's oral language development is shaped by the frequency and quality of their communicative interactions with caregivers (Hoff, 2006; Huttenlocher et al., 2010; Rowe, 2012). As such, a promising approach to promoting early language learning is to provide parents with activities, strategies and resources that scaffold and support rich communicative interactions in the home.

One strategy which has received considerable research attention is parent‐child shared book reading. Sharing books together, particularly when parents use strategies that encourage the child's active participation and interactive discussion of the book, exposes children to complex vocabulary and language structures and provides opportunities to practice using language, which boosts language growth (Hoff, 2006). Shared reading is consequently widely regarded as an effective method of early language intervention (Dowdall et al., 2020; Mol et al., 2008). However, several recent studies illustrate that shared reading interventions are not always effective (Lingwood et al., 2020; Noble et al., 2020) though programmes in those studies are typically of short duration. It has also been suggested that overall effects of shared reading interventions are small (Noble et al., 2019) and may be smaller for children from low SES backgrounds (Manz et al., 2010; Mol et al., 2008).

Potentially, supplementing dialogic reading approaches with more structured, direct support for language development may be more effective for those most in need. Vocabulary and narrative skills are common targets for early language intervention: These skills underpin oral language comprehension and reading comprehension and are critical for effective language use (Suggate et al., 2018); furthermore, they are often compromised in children from socially disadvantaged backgrounds (e.g. Dockrell, 2023; Levine et al., 2020). Interventions which target these aspects of language have been shown to improve children's language skills when delivered in school‐based settings (e.g. Dockrell et al., 2010; Fricke et al., 2017).

(Burgoyne et al., 2018) report findings from a randomised controlled trial of a 30‐week parent‐delivered intervention which supplements shared reading with targeted, explicit instruction on vocabulary and narrative skills. The intervention was evaluated with 208 preschool children (3–4 years) and their families, recruited through 22 Children's Centres (local community centres providing a range of free support services and resources to families with children from birth to 5 years) in areas of high socioeconomic deprivation. Families were randomly allocated to language intervention or an active treatment control targeting motor skills. The language intervention led to significant gains in 3–4‐year‐old children's language skills (*d* = 0.21) which were maintained 6‐months after intervention (*d* = 0.34). Children receiving language intervention also had better word reading (*d* = 0.35) and letter sound knowledge (*d* = 0.42) in the first year of school.

Whilst the findings of (Burgoyne et al., 2018) indicate positive effects of parent‐delivered early language intervention, context matters in evaluations of educational interventions such that outcomes and effect sizes often vary substantially between implementations (Coldwell & Moore, 2023; Lortie‐Forgues & Inglis, 2019). The challenges of implementing intervention at scale are noted: Increasing numbers of pupils and settings inevitably increases variability in implementation, and make it more difficult to provide the levels of support, monitoring and engagement that are possible in smaller trials. Implementation quality is an influential factor in the effectiveness of intervention programmes (Durlak & DuPre, 2008) including those targeting language (Rogde, Hagen, Melby‐Lervag, & Lervag, 2019). Other changes made in response to scaling up that can negatively impact effect sizes and lead to failure to replicate positive effects include changes to participants and recruitment methods, and adaptations to intervention (Maxwell et al., 2021). Thus it is important that further evaluation trials are conducted.

Here we sought to evaluate the parent‐delivered early language intervention reported in (Burgoyne et al., 2018) with a larger sample size. Since the earlier trial, the programme was revised and published by BookTrust (https://www.booktrust.org.uk/): Revisions included updating content to replace out of print books and reducing text instructions. Funding cuts to Children's Centres in the UK prompted contextual differences between trials, where families in the current trial were recruited and supported through school nurseries. Further, as detailed below, the trial was interrupted by the COVID‐19 pandemic resulting in adjustments to the protocol. Consequently, this study is not a direct replication of (Burgoyne et al., 2018). This paper focuses on a quantitative evaluation of the effectiveness of the programme; additional qualitative and implementation process data were collected via interviews and surveys completed with nursery staff and parents and are reported in the EEF evaluation report, available under the Open Government Licence (Menzies et al., 2022).

## METHOD

**FIGURE 1:**
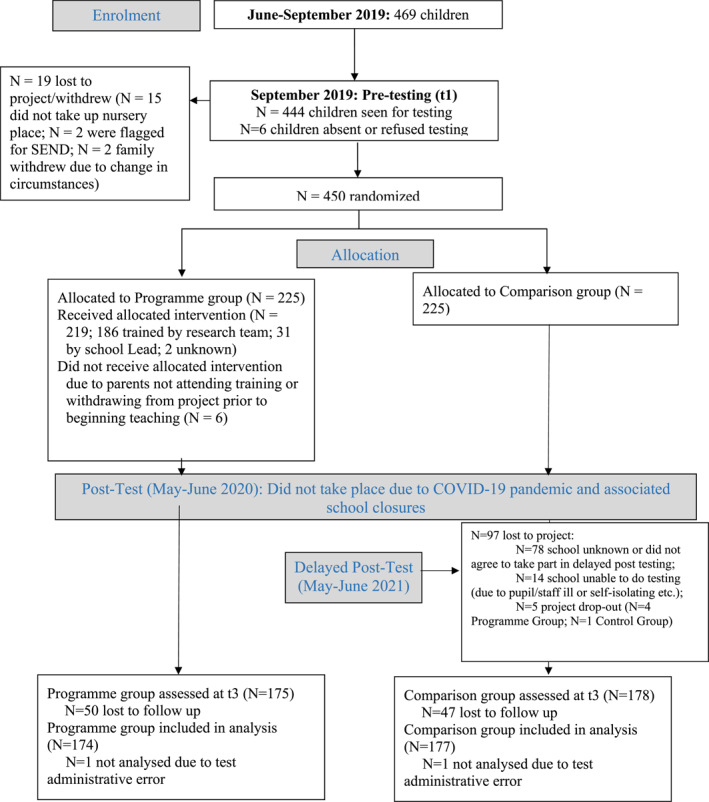
Consort diagram showing flow of participants through RCT study.

This randomised controlled trial (RCT) recruited 469 families from nurseries in Greater Manchester and Lancashire (UK) to evaluate the effectiveness of a parent‐delivered teaching programme targeting early language development in pre‐school children. The study was granted ethical approval by Durham University School of Education Ethics Committee. Informed parental consent was obtained for all children. Details of participant recruitment, allocation and flow through the study are summarized in the CONSORT diagram (Figure 1). This trial was pre‐registered with the ISRCTN registry (ISRCTN16848722; https://www.isrctn.com/ISRCTN16848772). The original protocol for the trial was published pre‐trial (Cramman et al., 2019); amendments due to COVID‐19 were published before data analysis (Cramman et al., 2021).

### Sample size

Sample size estimation for an effect size of *d* = 0.18, *p* < 0.05, 2‐tailed, pre‐post‐test correlation of *r* = 0.60, and 0.10 intracluster correlation (Xiao et al., 2016) reflected that *N* = 225 per arm will have 80% power. At post‐test, the study retained power to detect an effect size of *d* = 0.17.

### Participants

Forty‐seven state‐maintained nurseries and schools in Greater Manchester (29) and Lancashire (18) took part. Settings were recruited through Local Authorities supporting recruitment within their respective area, and information events to cascade project information to local settings.

Schools and nurseries recruited parents if they: (a) had a child in Nursery (aged 3–4 years), (b) were able to read and understand English, and (c) had no plans to move out of the area. Exclusion criteria were (a) twins or siblings in the same year group and (b) children with known or suspected learning difficulties or developmental disorders (e.g., autism). Settings were required to recruit a minimum of 4 families. In total 450 children were randomized and entered the study; the number of families per setting varied between 4 and 21.

Descriptive statistics for baseline pupil characteristics are presented in Table 1. Children were aged between 3 years, 0 months and 4 years, 1 month at pretest (mean age in years = 3.53). Parents reported that English was not the main language used at home for 38 children (8.44% of the sample; *N* = 19 in the PACT Programme group; *N* = 19 in the Comparison group). A fifth of the sample (22%; *N* = 99) scored at or below the 10th centile on at least two of three standardized language measures at pretest (BPVS and CELF Expressive Vocabulary and Sentence Structure) and could be described as having clinically significant language difficulties; however, English was not the main language used at home for 22 of these children for whom low scores may reflect inexperience with English. Being new to the setting and/or lack of familiarity with the researcher may also have impacted on some children's scores.

**TABLE 1:** Pupil level demographic and baseline characteristics.

Variables at baseline *(t1)*	Intervention group	Control group
	*N* (%)	*N* (%)
Gender	224 (100)	225 (100)
Female	98 (43.8)	104 (46.2)
Male	126 (56.2)	121 (53.8)
English is main language spoken at home	225 (100)	225 (100)
No	19 (8.4)	19 (8.4)
Yes	206 (91.6)	206 (91.6)
Early years pupil premium	215 (100)	220 (100)
No	185 (86.0)	180 (81.8)
Yes	30 (14.0)	40 (18.2)
Pre‐test completion status	225 (100)	225 (100)
Completed all	211 (93.8)	211 (93.8)
Partially completed	10 (4.4)	12 (5.3)
Did not complete	4 (1.8)	2 (0.9)

	*N*	Mean (SD)	*N*	Mean (SD)
Age in months	222	42.38 (3.45)	225	42.29 (3.45)
Language latent variable (baseline)	221	−0.03 (1.85)	223	0.03 (1.80)
CELF_SS (scaled score)	221	7.81 (2.88)	221	7.76 (3.19)
CELF_EV (scaled score)	217	8.80 (3.32)	218	8.80 (3.42)
Listening comprehension (raw score)	217	0.95 (1.06)	218	1.22 (1.19)
Receptive vocabulary (BPVS‐3; std. score)	214	90.33 (14.45)	214	90.39 (13.70)
APT information (raw score)	219	18.77 (7.05)	217	19.38 (6.39)
APT grammar (raw score)	219	13.56 (6.43)	217	14.64 (6.53)
Home learning environment index	186	28.87 (8.66)	187	28.91 (9.53)

Abbreviations: APT, Action Picture Test; EV, Expressive Vocabulary; SS, Sentence Structure.

Socioeconomic indicators were eligibility for Early Years Pupil Premium (EYPP) as reported by nursery settings (15.56% of the sample eligible) and home postcodes ranked using the English Indices of Deprivation (2015), where group 1 = ‘most deprived’ and 10 = ‘least deprived’. Nearly half (45.08%) of participants (*N* = 437; missing data: *N* = 9; participant withdrawals: *N* = 4) lived in group 1–2 postcodes, with 7.09% in group 9–10. Further details on settings and participant sample characteristics can be found in online Appendix A.

Within each setting children were randomly allocated to either the Programme Group (*N* = 225), where families received the language programme, or the Comparison Group (*N* = 225), where families received a box of storybooks at the end of nursery. The theoretical possibility of contamination effects is deemed highly unlikely as programme engagement depended upon an extensive set of resources (some non‐reusable) delivered to Programme group families during the intervention phase. During training, Programme Group parents/carers were told the importance of group allocation and instructed not to share materials. Implementation and process evaluation (Menzies et al., 2022) confirmed that contamination between groups was minimal. Group allocation was conducted independently by the Durham University research team at a single time point using a permuted block randomization scheme, and was balanced across groups for ability to complete pretests. Randomisation also aimed to ensure equal numbers of intervention and control group participants in each setting.

### Parent‐delivered early language teaching programme

The study evaluated a parent‐delivered early language teaching programme for 3–4 year old children. The programme originally developed and evaluated by (Burgoyne et al., 2018) was updated and published by BookTrust (https://www.booktrust.org.uk/) for the current trial. The programme aims to promote language development through interactive book reading, supplemented with direct teaching of vocabulary and work on narrative skills. The 30‐week programme is comprised of 5‐week teaching ‘blocks’, which are linked to topics (e.g., animals; the body). Each block is organised into 4 weeks of new learning material, with the final (5th) week dedicated to revision and extension activities. Teaching is designed to be delivered in 20 min sessions, 5 days per week (i.e., 150 sessions; 50 h of teaching in total) and is supported by teaching plans and resources.

The programme was developed with reference to Early Years policy and practice guidelines and in consultation with Early Years education professionals and speech and language therapists. Each teaching session follows a structured framework using short, varied activities to encourage and support engagement, consisting of: *Interactive book reading*: Shared reading of storybooks, using prompts and supports to encourage children's active participation; *Vocabulary teaching*: Targeted and structured teaching of vocabulary following principles of multiple context learning (Beck et al., 2013) and using visual supports and active learning strategies; *Storytelling*: Developing narrative skills through sequencing, summarizing, and retelling stories. An overview of the teaching programme and an example of a teaching session is provided in (Burgoyne et al., 2018).

### Programme training and support

One or two staff members from each setting were trained to recruit and support parents to deliver the programme and act as main school contact. School staff attended 1‐day training focusing on the programme materials but also covering project background and design, recruiting and supporting families, and data collection procedures. School staff were responsible for distributing intervention packs to families, and for providing support and encouragement to families throughout programme delivery: Recommendations for the types and frequency of setting‐level support were outlined but in practice, provision was determined by each setting.

Programme Group families (*N* = 225) were invited to a researcher‐delivered, small‐group training session at a local nursery lasting 1.5–2 h. Training included a brief overview of the project background and data collection procedures, but largely focused on explaining the teaching programme and modelling delivery. The majority of families (*n* = 186) attended this training; non‐attendees were subsequently trained by their school lead (using the same training materials; *n* = 31). Details of training were not recorded for 2 families who later started the programme, whilst 6 families did not attend any training or deliver any of the programme.

Programme group parents were asked to complete daily record forms using a digital app or paper record forms which recorded completion and enjoyment of each session.

## IMPACT OF COVID‐19

The trial took place between September 2019 and July 2021. COVID‐19 lockdown measures first legally came into force in the UK in March 2020. This caused significant disruption to intervention delivery including to family routines and parent delivery of the intervention, and delayed provision of the final 5‐weeks of programme materials. The trial protocol was disrupted as it was not possible to collect immediate post‐test data in summer 2020. Restrictions on social contact remained at delayed post‐test (summer 2021) such that planned researcher‐delivered assessments could not be completed. These were instead replaced by an alternative measure delivered by school staff. It was not possible to assess literacy skills at delayed post‐test as originally planned.

### Assessments

Children were assessed before randomization (September 2019; pretest, *t1*), and 10 months after intervention ended (June‐July 2021; delayed post‐test, *t3*) at which point children were nearing the end of Reception year. The original trial protocol included an immediate post‐test (*t2*) at the end of the intervention period (June‐July 2020) which could not be completed due to school closures associated with the COVID‐19 pandemic. Only the parent‐completed HLE questionnaire was completed at *t2*. Parents received a £10 gift voucher on completion of each child‐assessment.

At *t1*, children were assessed by the research team in their nursery using standardized language measures. In the original trial protocol these assessments were repeated at *t2* and *t3*; however, due to ongoing restrictions, *t3* data collection changed to an assessment completed by school staff.

#### Language assessments (*t1*)

*Expressive and Receptive Vocabulary*: Expressive vocabulary was measured using the CELF Preschool II^UK^ *Expressive Vocabulary* subtest (Semel et al., 2006) and the *Information Score* from the Renfrew Action Picture Test (4th Ed.) (APT; Renfrew, 2010). Receptive vocabulary was assessed using the *BPVS3* (Dunn et al., 2009).

*Expressive and Receptive Grammar:* Receptive grammar was measured using the CELF Preschool II^UK^ *Sentence Structure* subtest; the *Grammar Score* from the APT provided a measure of expressive grammar.

*Listening Comprehension:* Children listened to a short story adapted from the York Assessment of Reading for Comprehension (YARC; Hulme et al., 2009) and answered 8 questions about it.

#### Language assessments (*t3*)

*LanguageScreen* is a computerized language assessment with 4 subtests measuring expressive and receptive vocabulary, sentence repetition, and listening comprehension (https://www.languagescreen.com/). School staff completed the assessment with individual children. Scoring is automated and uploaded to secure servers.

#### Home Learning Environment (t1, t2)

The Home Learning Environment (HLE) Index (Melhuish et al., 2008) asks parents/carers to report the frequency (on a 0 to 7 scale) of seven routine activities including reading, library visits, and learning letters and numbers (max score 49). As COVID‐19 restrictions were in place at *t2*, the library visits item was removed (max score 42).

#### School readiness (t3)

The Brief Early Skills and Support Index (BESSI; https://www.cfr.cam.ac.uk/tests‐questionnaires/bessi) is a 30‐item questionnaire (with responses on a four point (strongly agree to strongly disagree) scale) completed by school staff, which assesses how well nursery and Reception children are making the transition to school.

### Statistical analysis

Analysis followed the pre‐registered plan (Kasim et al., 2021). The primary outcome measure was a language latent variable defined by four *LanguageScreen* subtests assessed at *t3* (expressive vocabulary, receptive vocabulary, recalling sentences, and listening comprehension). An Intention to Treat (ITT) principle was applied to analysis of outcomes using RStudio (RStudio Team, 2020), where a multilevel model (MLM) adjusted for prior attainment (baseline latent language variable including CELF Expressive Vocabulary and Sentence Structure, BPVS III, APT information, and Listening Comprehension) and accounted for variability in pupil attainment and intervention effects across schools.

Latent variables were constructed applying Confirmatory Factor Analysis in Mplus 7.4 (Muthen & Muthen, 1998‐2016) with Full Information Maximum Likelihood estimators to allow for missing data. Secondary outcome measures were scores on individual LanguageScreen subtests, the HLE at *t2*, and BESSI at *t3*.

Dosage‐response was investigated using Complier Average Causal Effect (CACE) analysis to explore the relationship between primary outcome and adherence to the intervention (number of intervention sessions reported as completed).

## RESULTS

At pretest (*t1*) we obtained data from 444 children, 351 of whom were tested at delayed posttest (*t3*) (i.e., overall attrition = 22%). Rates of attrition were essentially identical between the intervention and control group (Figure 1). A logistic regression assessed whether pupil characteristics varied by missingness at *t3*. There was no evidence that missing data varied by pupil characteristics or that pupil characteristics could predict drop out (Menzies et al., 2022).

Pretest data was complete for 422 children; partial data was obtained for 22 children. Six children did not complete pre‐testing due to absences/non‐compliance (see Table 1).

Programme group families reported completing an average 87.19 intervention sessions out of a possible 150 (standard deviation = 51.32, reflecting a wide range of 0–150 sessions completed), equating to an average 17.4 weeks completed.

### Primary outcome

Descriptive statistics (means and standard deviations) on primary and secondary outcome measures are reported by group in Table 2 along with intervention effect sizes.

**TABLE 2:** Mean scores (SD) on trial primary and secondary outcome measures with effect sizes for intervention effects by group.

	PACT programme		Control group		Effect size (hedges' g) [95% CI]
	*N* = 225		*N* = 225		
	*N*	Mean (SD)	*N*	Mean (SD)	PACT versus control
Primary outcome					
LanguageScreen latent variable (t3)	174	−0.02 (9.60)	177	0.02 (10.20)	0.01 (−0.27, 0.31)
Secondary outcomes					
LanguageScreen_EV (t3)	174	105.98 (13.27)	177	105.18 (14.27)	0.08 (−0.20, 0.36)
LanguageScreen_RV (t3)	174	104.71 (13.96)	177	104.93 (13.91)	0.04 (−0.22, 0.31)
LanguageScreen_LC (t3)	174	105.57 (14.33)	177	106.65 (14.23)	−0.06 (−0.34, 0.21)
LanguageScreen_SR (t3)	174	101.87 (13.24)	177	102.45 (13.96)	−0.05 (−0.30, 0.20)
Home learning environment					
HLE index *(t2)*	137	28.37 (7.83)	168	27.90 (9.10)	0.10 (−0.15, 0.34)
School readiness					
BESSI (t3)	170	3.24 (4.09)	169	3.17 (4.11)	−0.03 (−0.26, 0.19)

Abbreviations: BESSI, Brief Early Skills and Support Index; EV, Expressive Vocabulary; HLE, Home Learning Environment; LC, Listening Comprehension; RV, Receptive Vocabulary; SR, Sentence Repetition.

**FIGURE 2:**
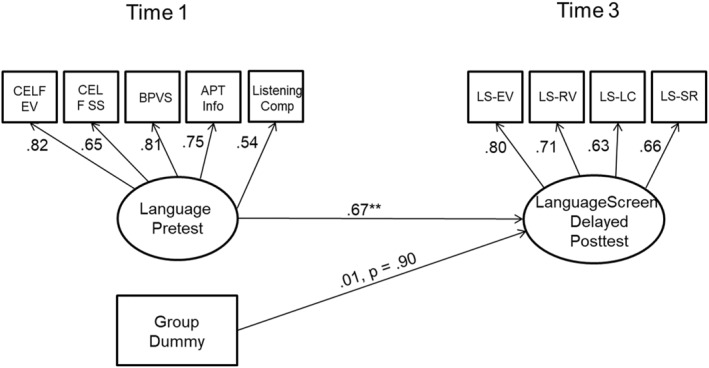
Model results for pre‐test and post‐test latent variables used in the analysis of primary outcome.

Our primary outcome was a language latent variable defined by the four LanguageScreen Subtests (i.e., Receptive Vocabulary, Expressive Vocabulary, Listening Comprehension and Sentence Repetition) at delayed post‐test (*t3*). Such a measure assesses an underlying factor that captures the common variance shared by the different language subtests. The models used are shown in Figure 2 and provide excellent fits to the data (baseline (*t1*): *χ* ^2^ (5) = 9.44, *p* = 0.09; RMSEA (Root Mean Square Error of Approximation) = 0.045 [90% CI 0.00, 0.08]; CFI = 0.99; TLI = 0.99; delayed post‐test (*t3*): *χ* ^2^ (2) = 2.97, *p* = 0.23; RMSEA = 0.037 [90% CI 0.00, 0.12]; CFI = 0.99; TLI = 0.99). Values of RMSEA <0.06 and CFI >0.95 are considered indicative of acceptable model fit.

The two language latent variables, based on different assessments completed 21 months apart, were reasonably correlated (*r* = 0.67). The most critical result from these analyses is that there is no evidence of group differences in language ability at delayed post‐test (*g* = 0.01 [95% CI −0.27, 0.31]).

### Secondary outcomes

#### LanguageScreen subtests

The standardised LanguageScreen subtest scores (Table 2) were used to investigate whether there was improvement in particular aspects of language. Each of the models analysing secondary outcomes on the LanguageScreen subtests used the pre‐test language latent variable as the baseline measure for effect size estimation. This ensured a consistent approach to mitigate the fact that the study required different measures at pre‐ and post‐test. There was moderate correlation between the pre‐test latent variable and LanguageScreen sub‐tests: RV, *r* = 0.67; EV, *r* = 0.63; LC, *r* = 0.40; SR, *r* = 0.42. No significant group differences were observed for any of the LanguageScreen subtests: effect sizes (*g*) were small ranging between 0.08 for expressive vocabulary and −0.06 for listening comprehension. Thus, there was no evidence that the teaching programme led to significantly greater language skills when assessed 10 months after teaching ended.

#### Home Learning Environment (HLE)

The HLE Index (*t1*) was used as pre‐test score when analysing post‐test (*t2*) HLE Index as a secondary outcome; the correlation between these variables was *r* = 0.50. There was no evidence that the intervention group had higher HLE scores than the control group at immediate post‐test (*g* = 0.10 [95% CI −0.15, 0.34]).

#### School readiness (BESSI)

The pre‐test language latent variable was used as the baseline measure for effect size estimation in the analysis of BESSI scores at delayed post‐test (*t3*); these variables were moderately correlated (*r* = 0.40). There were no group differences on this measure (*g* = −0.03 [95% CI −0.26, 0.19]).

#### Dosage‐response

CACE sensitivity analysis found a positive association between effect size and dosage level, indicating that the effect size would be higher with increased dosage. However, whereas the intervention group had an average of 58% compliance it was observed that even at high levels of dosage (more than 80% of intervention sessions completed) effect sizes remained low and uncertain (CACE = 0.05 [95% CI −0.31, 0.45]).

## DISCUSSION

This paper reports findings from a large‐scale, pre‐registered trial which aimed to evaluate the effectiveness of a parent‐delivered early language teaching programme, previously shown to improve children's early language and literacy skills (Burgoyne et al., 2018). Significant disruptions to the trial were caused by the COVID‐19 pandemic and consequently it was not possible to collect language outcome data immediately after teaching ended (*t2*). Language assessments completed 10 months later (*t3*) found no evidence that the programme led to gains in children's language skills and no evidence that the programme impacted on school readiness or the HLE.

These findings are potentially important, but are nonetheless difficult to interpret, particularly given the contrast with findings from the previous trial which reported significant gains on measures of early language assessed immediately after intervention (*d* = 0.21) and larger effects of intervention 6‐months later (*d* = 0.34) (Burgoyne et al., 2018). Here we consider potential explanations for differences in trial findings, drawing on issues discussed in implementation science.

Arguably the most obvious explanation is the impact of COVID‐19 on the current trial. Most significantly, as it was not possible to collect immediate post‐test data it is unknown whether the intervention had a significant impact on language outcomes immediately following intervention. Language outcome data was only collected 10‐months after intervention ended, when children were at the end of Reception year. Whilst it may be reasonable, based on previous trial findings, to expect that any delayed effects would be observable at this point, it is important to note that the implementation context changed significantly during the trial as a result of COVID‐19 (Coldwell & Moore, 2023), including the national roll‐out of early language intervention in schools for children in Reception year (https://www.gov.uk/government/news/every‐school‐with‐reception‐class‐offered‐early‐language‐training). Children in the study may therefore have benefited from this school‐based language intervention between pre‐and post‐testing.

Also as a result of COVID‐19 and continuing restrictions on social interaction, the language assessment at delayed post‐test (LanguageScreen) differed from the assessments at baseline. The correlation between pre‐ and post‐test language latent variables, assessed 21 months apart, was therefore not surprisingly weaker in the current trial (*r* = 0.67) than in the previous trial (*r* = 0.92 at delayed post‐test) where the measures used were the same at each time point. Potentially LanguageScreen may not have been sensitive enough to capture changes in language skills in this study, though it has been shown to be sensitive to effects of comparable language intervention (e.g., West et al., 2021).

Beyond COVID‐19, there are other potential explanations for the lack of effects seen in the current study. Contextual differences between trials may have contributed to the failure to replicate positive effects (Maxwell et al., 2021), including changes to recruitment and sample differences. Where the first trial worked with Children's Centres in low‐income areas to engage families who may be most able to benefit from this type of intervention, families in the current study were recruited and supported through maintained nurseries and schools. Whilst we made efforts to recruit settings from socially disadvantaged areas, there is considerable variability in the sample, with only 15.56% of children eligible for EYPP. Further, the sample as a group are slightly older than children in the previous trial, and though a proportion of children in the current study (22%) had apparent language difficulties, as a group they have better language skills than children in the previous trial and are not characterized by low language ability. Thus, many children and families taking part in the current study do not appear to be in need of intervention and may not have been best placed to benefit.

It is also worth noting that the programme materials were revised and adapted for publication prior to use in the current study; these adaptations may potentially have negatively impacted on outcomes (Maxwell et al., 2021). However, nursery staff and parents reported high levels of satisfaction with the programme and perceived benefits of the programme including improving children's language outcomes, increased enjoyment of reading books, and better readiness for school (Menzies et al., 2022). Though this is not in line with evidence from the assessment data, it adds to understanding of the intervention and suggests further evaluation is warranted.

It is also important to consider the influence of implementation variability (e.g., Durlak & DuPre, 2008). Families reported completing an average of 17.4 weeks of the programme, which is identical to the previous trial. However, weeks completed is a crude measure and parent‐reported data may not be reliable. Further, implementation and process evaluation (Menzies et al., 2022) suggests variability in fidelity to the programme with some parents reporting adaptations of the programme. At the setting‐level, support for families also varied widely and in some instances may not have been of the level needed to support implementation quality.

Attrition in this study was relatively high (22%) but is in line with the previous evaluation (24%; Burgoyne et al., 2018) and with other studies of parent delivered language interventions (e.g., Gibbard et al., 2024, p. 19%); importantly, rates of attrition were essentially identical between the two arms.

No significant effects of the language intervention were found on the HLE measured via parent survey immediately following intervention. This is surprising given that the intervention involved storybooks and additional activities and resources for parents and children to work on together at home. The COVID‐19 context may have impacted on the capacity to detect differences between groups as nurseries provided more activities and resources for parents to work on at home during lockdown (Menzies et al., 2022), potentially minimizing any differences between the programme and control group families. It should also be noted that there were high levels of missing data (32%) on this measure at post‐test.

Finally, no significant effects of intervention were found on school readiness (the BESSI) measured at the end of Reception Year. Similarly, West et al. (2024) found no effects of language intervention on this measure when assessed at the end of nursery. This measure may not be sensitive to group differences at this age; further studies may therefore benefit from using alternative measures to assess school readiness.

In summary, there was no evidence in this study that the parent‐delivered early language teaching programme evaluated here led to significant improvements in children's language abilities, school readiness or the HLE. These findings are however not conclusive. Plausible interpretations of these findings relate to the COVID‐19 context and more broadly to issues of implementation and contextual influences; further work under less challenging conditions is needed to reach reliable conclusions about the potential effectiveness of the programme.

## AUTHOR CONTRIBUTIONS

**Kelly Burgoyne**: Conceptualization; funding acquisition; investigation; methodology; project administration; supervision; writing—original draft; writing—review & editing. **Stephanie Hargreaves**: Investigation; project administration; writing—original draft. **Nasima Akhter**: Formal analysis; writing—original draft; writing—review & editing. **Helen Cramman**: Conceptualization; data curation; funding acquisition; investigation; methodology; project administration; supervision; writing—original draft; writing—review & editing. **Paivi Eerola**: Investigation; project administration; writing—original draft; writing—review & editing. **Jochen Einbeck**: Formal analysis; writing—original draft; writing—review & editing. **Victoria Menzies**: Conceptualization; data curation; funding acquisition; investigation; methodology; project administration; supervision; writing—original draft; writing—review & editing.

## CONFLICT OF INTEREST STATEMENT

The authors have declared no competing or potential conflicts of interest.

## ETHICAL CONSIDERATIONS

The study was granted ethical approval by Durham University School of Education Ethics Committee. Informed parental consent was obtained for all children.

## Supporting information

Supporting Information S1

Supporting Information S2

## Data Availability

Data availability statement: Partial data that supports the findings (excluding data from LanguageScreen assessment due to legal restrictions on use and sharing of the data) has been submitted to the Education Endowment Foundation's (EEF) Data Archive hosted by FFT (https://fft‐uat5‐ui.metadata.works/browser/landing%20[fft‐uat5‐ui.metadata.works). This data will be made available through application to the Education Endowment Foundation.
