# Supplementary material for: Lack of effect of a parent‐delivered early language intervention: Evidence from a randomised controlled trial completed during COVID‐19
Source: JCPP Adv. 2024 Sep 14;5(3):e12279. doi: 10.1002/jcv2.12279 (PMC12446704; doi:10.1002/jcv2.12279)
Supplement: Supplementary file 1 — Supporting Information S1 [file JCV2-5-e12279-s001.docx]

Online Appendix A: Sample characteristics

Table 1 Setting Level Characteristics

| School level variables (categorical) | National-level mean | All Schools (both Intervention and Control group as within school randomization design) | |
| --- | --- | --- | --- |
|  |  | Count | Percentage |
| School Ofsted rating |  |  |  |
| Outstanding |  | 14 | 29.8% |
| Good |  | 28 | 59.6% |
| Requires improvement |  | 3 | 6.4% |
| Inadequate |  | 0 | 0.0% |
| No data |  | 2 | 4.3% |
| School-level  Reading progress score (2019) |  | Count | Percentage |
| Well below average |  | 2 | 5.88% |
| Below average |  | 3 | 8.82% |
| Average |  | 24 | 70.59% |
| Above average |  | 3 | 8.82% |
| Well above average |  | 2 | 5.88% |
| Missing data |  | 13 |  |
| Type of school |  | Count |  |
| Local authority nursery |  | 9 | 19% |
| Community |  | 26 | 55% |
| Academy converter |  | 3 | 6% |
| Voluntary aided |  | 7 | 15% |
| Foundation |  | 1 | 2% |
| Voluntary Controlled |  | 1 | 2% |
| School location |  | Count | Percentage |
| Urban city and town |  | 18 | 38% |
| Urban major conurbation |  | 28 | 60% |
| No data |  | 1 | 2% |
| School level variable (continuous) | National Average | Mean (sd) |  |
| Free School Meals | 19.7% | 29.1% (17.5) |  |

Table 2 Pupil Level Characteristics

| Pupil characteristics | Intervention group | | Control group | |
| --- | --- | --- | --- | --- |
| Pupil-level  (categorical) | n/N  (missing) | Count (%) | n/N  (missing) | Count (%) |
| **Early Years Pupil Premium** | (10 missing) |  | (5 missing) |  |
| No | 185/215 | 86.0 | 180/220 | 81.8 |
| Yes | 30/215 | 14.0 | 40/220 | 18.2 |
| Pupil-level  (continuous) | n/N  (missing) | Mean (SD) | n/N  (missing) | Mean (SD) |
| **Attendance at Nursery (hours per week)** | 0 missing | 22.64 (8.34) | 0 missing | 22.37 (7.97) |
